# Structural interpretation of protein-protein interaction network

**DOI:** 10.1186/1472-6807-10-S1-S4

**Published:** 2010-05-17

**Authors:** Ataur R Katebi, Andrzej Kloczkowski, Robert L Jernigan

**Affiliations:** 1 L.H. Baker Center for Bioinformatics and Biological Statistics Iowa State University, Ames, Iowa 50011-0320, USA; 2 Bioinformatics and Computational Biology Iowa State University, Ames, Iowa 50011-0320, USA; 3 Department of Biochemistry, Biophysics and Molecular Biology Iowa State University, Ames, Iowa 50011-0320, USA

## Abstract

**Background:**

Currently a huge amount of protein-protein interaction data is available from high throughput experimental methods. In a large network of protein-protein interactions, groups of proteins can be identified as functional clusters having related functions where a single protein can occur in multiple clusters. However experimental methods are error-prone and thus the interactions in a functional cluster may include false positives or there may be unreported interactions. Therefore correctly identifying a functional cluster of proteins requires the knowledge of whether any two proteins in a cluster interact, whether an interaction can exclude other interactions, or how strong the affinity between two interacting proteins is.

**Methods:**

In the present work the yeast protein-protein interaction network is clustered using a spectral clustering method proposed by us in 2006 and the individual clusters are investigated for functional relationships among the member proteins. 3D structural models of the proteins in one cluster have been built – the protein structures are retrieved from the Protein Data Bank or predicted using a comparative modeling approach. A rigid body protein docking method (Cluspro) is used to predict the protein-protein interaction complexes. Binding sites of the docked complexes are characterized by their buried surface areas in the docked complexes, as a measure of the strength of an interaction.

**Results:**

The clustering method yields functionally coherent clusters. Some of the interactions in a cluster exclude other interactions because of shared binding sites. New interactions among the interacting proteins are uncovered, and thus higher order protein complexes in the cluster are proposed. Also the relative stability of each of the protein complexes in the cluster is reported.

**Conclusions:**

Although the methods used are computationally expensive and require human intervention and judgment, they can identify the interactions that could occur together or ones that are mutually exclusive. In addition indirect interactions through another intermediate protein can be identified. These theoretical predictions might be useful for crystallographers to select targets for the X-ray crystallographic determination of protein complexes.

## Background

Because of the use of high throughput experimental methods such as yeast two-hybrid screening [1], the number of reported protein-protein interactions (PPI) has increased dramatically. To extract meaningful information from this interaction data set, clustering of the interacting proteins is an established method. Patra *et al.*[2] have shown that functionally significant clusters can be extracted from the dominant eigenvalues of a modified contact matrix known as the Kirchhoff matrix. Sen *et al*. [3] used an eigenmode analysis (a type of spectral clustering) to cluster the interacting proteins.

The BioGrid database has published different versions of yeast protein interaction data with increasing numbers of proteins and interactions [4]. Some limited attempts have been made to construct spatial interaction clusters from this data. With early results showing that such clusters have functional relationships, such results may help to predict undiscovered interactions among proteins in the same cluster [3]. However the protein interaction data obtained from high-throughput screening methods such as the yeast two-hybrid method [1] and affinity purification techniques [5] are highly error-prone. Approximately, 30–60% false positives and 40–80% false negatives have been estimated for these methods [6,7]. Therefore predicting new interactions or drawing any conclusions from this interaction dataset requires validation of the interactions. Another complementary source of information about the proteins is their individual structures. If there were sufficient known structures of the protein-protein pairs they could provide direct validation of the interactions. However, the number of such known structures remains small, and certainly nowhere near the number of interacting pairs that have been reported. But there are relatively large numbers of individual protein structures. Those, together with improvements in docking methods make it possible to begin investigating the likelihood of forming individual three dimensional pairs of structures [8]. Looking at the 3D structure of each protein, especially the binding sites, in an interacting cluster can reveal information that can aid in validating the pair-wise interactions. Some questions that we set out to investigate here are:

1. Whether two proteins prefer to interact?

2. If more than two proteins purportedly interact with the same protein, can they interact concurrently by binding two separate regions of the protein, or does one exclude the other because their binding sites substantially overlap?

3. What are the relative binding strengths of proteins within a cluster?

We choose the yeast protein-protein interaction network from the online database BIOGRID(http://www.thebiogrid.org) [4]. The number of distinct proteins and interactions in the dataset has increased manyfold since the analysis by Sen *et al*. [3]. The current dataset (version 2.0.55) has over five thousand proteins and more than 145,000 interactions.

## Methods

We applied an eigenmode analysis to cluster the protein interaction network. We formed the Kirchhoff matrix [2] M; the interaction matrix M:(1)

Then, we performed eigenmode analysis of this matrix M. This definition automatically leads to a singular matrix (i.e. the determinant of the matrix M is zero) that must be analyzed with Singular Value Decomposition [3].

### Singular value decomposition (SVD)

We calculated all eigenvalues and eigenvectors of the connectivity matrix by applying the SVD subroutine available in the LAPACK library [9].

If A is any matrix of size m×n (with m>=n), then A can be written as a product of three matrices:

A = UΛV^T^						(2)

where Λ is the square matrix of size n×n containing nonnegative values λ_1_, λ_2_ , … ,λ_n_ along the diagonal and zeros off diagonal, and U and V are two matrices of sizes m×n and n×n, respectively, having orthogonal columns, i.e.(2)

and(3)

The Kirchhoff matrix M can be written as

M = VΛU^T^      						    	(4)

where Λ is the diagonal matrix containing eigenvalues λ_1_, λ_2_ , … ,λ_n_ of M and U is the matrix formed from eigenvectors of M. Thus, the elements M_ij_ of the contact matrix M can be expressed as(4)

where u_ki_ denotes the i^th^ component of the eigenvector corresponding to the k^th^ eigenvalue. Equation 4 is the eigenvalue expansion of the contact matrix. From Equation 5, it follows:(5)

The eigenvalues with the smallest indices corresponding to the largest absolute values of λ make the largest contributions and smaller eigenvalues contribute successively less [3].

### Cluster formation

For each eigenvalue there is a corresponding eigenvector. The significant components of an eigenvector comprise a cluster where each component corresponds to one protein. The components with an absolute value greater than 0.05 are assumed to be significant [3]. The clusters for larger eigenvalues are thus the interesting ones.

### Interaction complex formation within a cluster

**Figure 1:**
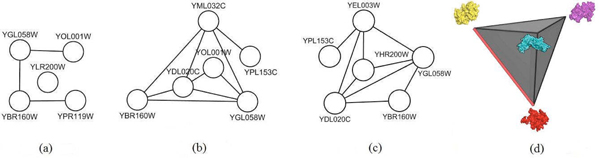
**Examples of three clusters and their interactions with the nodes being the proteins and their names given** (a) cluster 10 (b) cluster 14 (c) cluster 15 (d) New core of cluster 14 after YML032C and YPL153C were removed is shown schematically with yellow being YBR160W, purple YGL058W, cyan YDL020C, and red YOL001W. All 6 edges of this tetrahedron correspond to pairs of proteins that interact, with the red edge being the newly proposed interaction.

**Figure 2:**
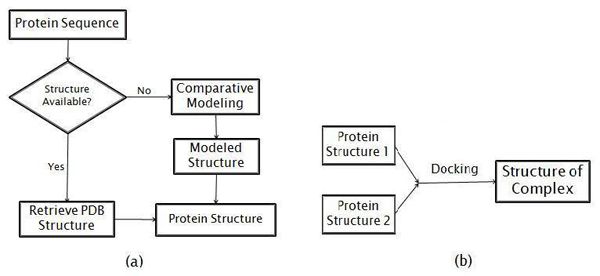
**Method for structure prediction of a protein interaction pair** (a) Flowchart for obtaining each protein structure (b) Flowchart for docking two proteins to form a docked complex

After the clusters are constructed, we need to choose a cluster to do structural analysis. Figure 1 shows three representative clusters (10, 14, and 15) for their moderate size. Out of these, we chose cluster 14 for further structural analysis. Then we attempt to predict the interaction complexes, predict new interactions, and predict whether multiple interactions could occur concurrently. The steps of this process are shown in the flowchart in Figure 2. In part 2(a) of the figure, an interacting partner protein structure is either retrieved from the Protein Data Bank (PDB) [10] (http://www.rcsb.org), or if there is no structure of the protein we predict the structure by comparative modeling. Figure 2(b) shows that once we have both structures of a putative interacting pair, we then use docking to predict the structure of the interaction complex.

###  Comparative modeling

To predict an interaction complex or predict a new interaction, we require the protein structures of both interacting proteins. If the structure of a protein is not available in the PDB, we use comparative modeling approaches [11,12]. To predict the structure of the protein, we have relied upon Zhang’s I-TASSER server [12-14] (http://zhanglab.ccmb.med.umich.edu/I-TASSER/), which gave the best protein models at the Critical Assessment of Structure Prediction (CASP 7 and CASP 8), a community-wide, worldwide experiment designed to obtain an objective assessment of the state-of-the-art in structure prediction [15-17]. The I-TASSER algorithm consists of three consecutive steps: threading, fragment assembly, and iteration. During threading, I-TASSER generates the template alignments by a simple sequence Profile-Profile Alignment approach constrained with the secondary structure matches. Fragment assembly is performed on the basis of threaded alignments and the target sequences are divided into aligned and unaligned regions. The fragments in the aligned regions are used directly from the template structures and the unaligned regions are modeled with ab initio simulations. Clusters of decoys are generated with the use of a knowledge-based force field. The cluster centroids are generated by averaging the coordinates of all clustered decoys and ranked based on the structure density. In the iteration phase, the steric clashes of the cluster centroids are removed and the topology is refined. The conformations with the lowest energy are selected.

 The I-TASSER server returns the best five models with a c-score attached for each model. Also it returns the top ten templates used in the threading. The c-score is a confidence score that I-TASSER uses to estimate the quality of the predicted model. The calculation of c-score is based on the significance of the threading template alignments and the convergence parameters of the structure assembly simulations. When selecting one of these models, we select the model that comes from the largest cluster and has the best c-score. C-score is in the range [5,2], where a higher c-score value signifies a better model [14].

### Docking

After we have both structures in an interacting pair we use docking to predict the protein complex formed in a protein-protein interaction. We use the Cluspro server [18-23] for docking the interacting proteins to predict the protein complex. Cluspro is the first fully automated web-based program for docking proteins and was one of the top performers at CAPRI (Critical Assessment of Predicted Interactions) rounds 1-12, the community-wide experiment devoted to protein docking [24]. The Cluspro server is based on a Fast Fourier Transform correlation approach, which makes it feasible to generate and evaluate billions of docked conformations by simple scoring functions. It is an implementation of a multistage protocol: rigid body docking, an energy based filtering, ranking the retained structures based on clustering properties, and finally, the refinement of a limited number of structures by energy minimization. The server (http://cluspro.bu.edu/) returns the top models based on energy and cluster size. We select one of the returned models after considering the energy and the size of the cluster – preferring lower energies and larger cluster sizes. As the Cluspro server implements rigid body docking, when a partner protein in a complex is structurally flexible Cluspro is not so able to account for this flexibility.

## Results

We perform the eigen-analysis on the yeast network version 2.0.40 (5,226 proteins and 114,754 interactions) and 2.0.41 (5,425 proteins and 121,664 interactions) and find that the number of zero eigenvalues are 6 and 3, respectively, which are very small compared to those from the yeast network Sen et al. previously used [3] (4,906 proteins, 19,037 interactions, and number of zero eigenvalues 46). This decrease in the number of zero eigenvalues is an indication of the completeness of the yeast network.

The proteins and their interactions in clusters 10, 14, and 15 are shown in Figure 1. We note that the number of neighbors for each protein in each of these clusters falls within a relatively small range. Those ranges are 278 – 288 for the proteins in cluster 10; 261 – 286 for the proteins in cluster 14; and 265 – 286 for the proteins in cluster 15.

We search the gene ontology database [25] for the functions of the proteins in each cluster and find that the proteins in each cluster have related functions usually. This is consistent with previous findings [3,26]. Table 1 shows the functions of each of the proteins in clusters 10, 14, and 15. The majority of the proteins in clusters 10 and 14 are cell cycle related; while cluster 15 is related to protein folding and protein degradation. We also attempt to determine the statistical confidence regarding the functional coherency of the clusters. We used FunSpec(http://funspec.med.utoronto.ca/) [27], a web based cluster interpreter for yeast, to measure the functional coherency of the clusters (see Table 2). FunSpec assesses the degree of functional enrichment for a given cluster by the hypergeometric probability distribution[28]. For each cluster, the probability (p-value) of observing such an overlap by chance is calculated as:(6)

**Table 1:** Functions of proteins in clusters 10, 14, 15 of yeast protein network-2.0.41

Protein name	Function	Function type
Cluster 10		

YBR160W	Catalytic subunit of the main cell cycle cyclin-dependent kinase	***Cell cycle***
YGL058W	Ubiquitin-conjugating enzyme (E2), involved in postreplication repair (with Rad18p), sporulation, telomere silencing, and ubiquitin-mediated N-end rule protein degradation (with Ubr1p)	Protein repair/ degradation
YLR200W	Subunit of the heterohexameric Gim/prefoldin protein complex involved in the folding of alpha-tubulin, beta-tubulin, and actin	Protein folding
YOL001W	Cyclin, negatively regulates phosphate metabolism	***Cell cycle***
YPR119W	B-type cyclin involved in cell cycle progression	***Cell cycle***

(b) Cluster 14		

YBR160W	Catalytic subunit of the main cell cycle cyclin-dependent kinase	***Cell cycle***
YOL001W	Cyclin, negatively regulates phosphate metabolism	***Cell cycle***
YPL153C	Protein kinase, required for cell-cycle arrest in response to DNA damage	***Cell cycle***
YML032C	Stimulates strand exchange by facilitating Rad51p binding to single-stranded DNA	***Cell cycle***
YDL020C	Transcription factor that stimulates expression of proteasome genes Type	Protein degradation
YGL058W	Ubiquitin-conjugating enzyme (E2), involved in postreplication repair (with Rad18p), sporulation, telomere silencing, and ubiquitin-mediated N-end rule protein degradation (with Ubr1p)	Protein repair/ degradation

(c) Cluster 15		

YGL058W	Ubiquitin-conjugating enzyme (E2), involved in postreplication repair (with Rad18p), sporulation, telomere silencing, and ubiquitin-mediated N-end rule protein degradation (with Ubr1p)	Protein repair/ degradation
YBR160W	Catalytic subunit of the main cell cycle cyclin-dependent kinase	***Cell cycle***
YEL003W	Subunit of the heterohexameric cochaperone prefolding complex which binds specifically to cytosolic chaperonin and transfers target proteins to it	Protein folding
YDL020C	Transcription factor that stimulates expression of proteasome genes Type	Protein degradation
YHR200W	Non-ATPase base subunit of the 19S regulatory particle (RP) of the 26S proteasome	Protein degradation
YPL153C	Protein kinase, required for cell-cycle arrest in response to DNA damage	***Cell cycle***

**Table 2:** MIPS functional classification and GO(Gene Ontology) assignments of biological processes and molecular functions for clusters 10, 14, and 15

Cluster #	# proteins	GO molecular function	GO biological process	MIPS functional classification
10	5	Cyclin-dependent protein kinase regulatory activity (5×10^-5^ ) Tubinding (4×10^-3^)	Regulation of cyclin-dependent protein kinase activity (6×10 ^-5^) Negative regulation of phosphate metabolic process (9×10^-4^)	Enzymatic activity regulation / enzymeRegulator (5×10^-4^) Regulation of phosphate metabolism(9×10^-3^)

14	6	Recombinase activity ( 2×10^-3^) DNA strand annealing activity ( 3 ×10^-3^)	Postreplication repair ( 1×10^-4^)regulation of cell cycle (5×10^-4^) Response to DNA damage stimulus (7 × 10^-4^)	DNA repair (3×10^-4^) G2/M transition of mitotic cell cycle (7×10^-4^)

15	6	Protein serine/threonine/tyrosine kinase activity (5×10^-3^)	Regulation of cell cycle (6×10^-4^)Negative regulation of meiotic cell cycle (10 ×10^-4^)	Proteasomal degradation (ubiquitin/proteasomal pathway) (2×10^-4^ )

where, G = the size of the genome; C = the number of genes in the genome having that attribute; n = the size of the query cluster; k = the number of genes in the cluster known to have that attribute [28].

Most of the p-values in Table 2 are quite small (< 10^-3^) for the three clusters we are reporting here. These small p-values signify the relatively strong functional coherency of these clusters. How small must a p-value be in order for a cluster to be functionally coherent? FunSpec uses 0.01 as a cut off, which is arbitrary. For each of the clusters, we obtain p-values that are much smaller than 0.01, indicating the highly probable functional coherency of the clusters.

One of our goals in this paper is to test the validity of a reported interaction by using structural information about the interacting proteins in a cluster. Our idea is simple: first, find the structures of the two interacting proteins from the PDB [10]. If the experimental structure is not available in the PDB for any of the proteins, we predict its structure by comparative modeling. For comparative modeling, we used both CABS modeling [11] and I-TASSER [12-14]. However, the results shown here come only from using I-TASSER. Once, we have both structures, we dock them to predict the interaction complex. We can repeat this method to verify individual interaction in a cluster.

**Figure 3:**
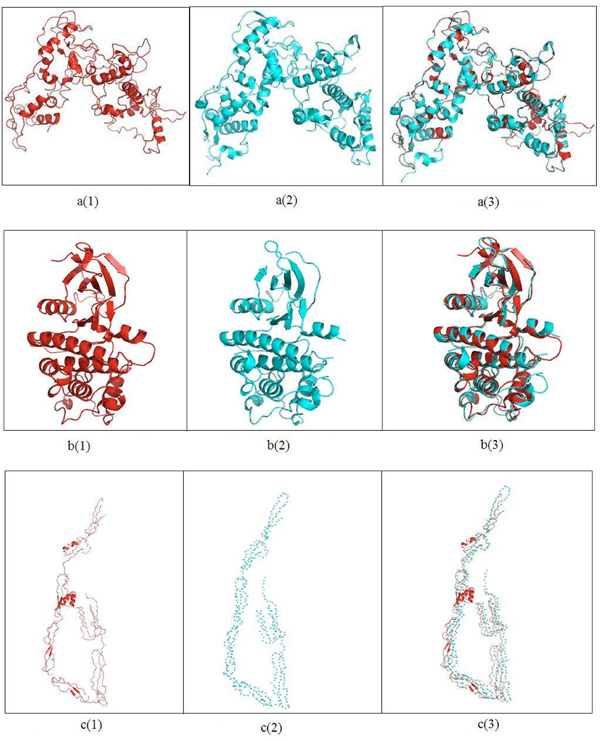
**Comparative modeling for three unknown proteins in cluster 14 shown in Figure 2** a(1) Model for YDL020C a(2) One of the templates used by I-TASSER (PDB ID:1Z1NX) a(3) Superimposition of the model and the template (RMSD = 0.410)  b(1) Model for YBR160W b(2) One of the templates used by I-TASSER (PDB ID:2PK9A) b(3) Superimposition of the model and the template (RMSD = 0.77) c(1) Model for YML032C c(2) Template used by I-TASSER (PDB ID:1WORA ) c(3) Superimposition of the model and the template (RMSD = 0). The difference in buried surface area for the model in a(1) and template in a(2) is 173 Å^2^ and that is in b(1) and b(2) 654 Å^2^.

**Figure 4:**
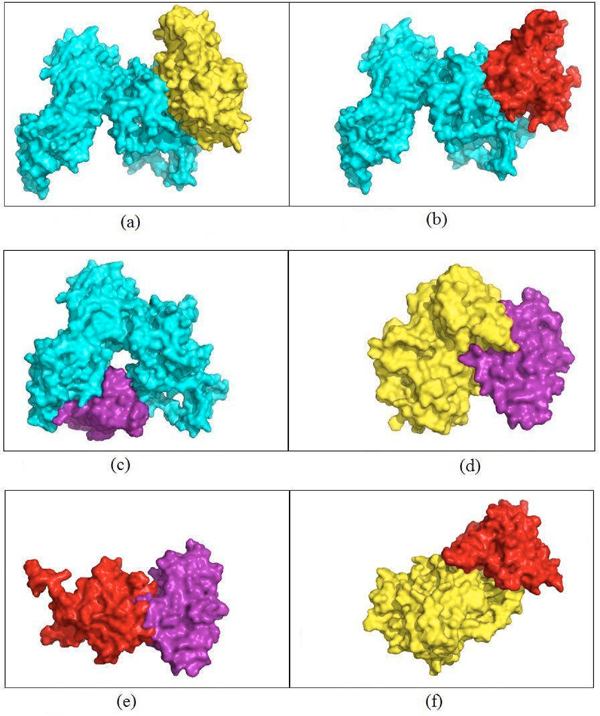
**Models built for the interactions in the core of cluster 14** Buried surface areas of the dimers (a) YDL020C.YBR160W(5,603Å^2^) (b) YDL020C.YOL001W (4,517Å^2^) (c)YDL020C.YGL058W(4,295Å^2^) (d)YBR160W.YGL058W(3,408Å^2^) (e)YOL001W.YGL058W(2,162 Å^2^)  (f)YBR160W.YOL001W (5,779 Å^2^).

Here, we show an example of this approach. We find the homologs for the six proteins in cluster 14 shown in Figure 1. For the three proteins – YOL001W, YPL153C, and YGL058W – we retrieve the PDB structures having 100% identity as 2PK9 chain B, 1QU5 chain A, and 1AYZ chain A, respectively. For the other three proteins – YBR160W, YML032C, and YDL020C – the PDB homologs are 3EZR chain A (62% identity), 1KN0 chain A (53% identity), and 1A1I chain A (43% identity), respectively. For the latter three proteins, we predict their structures using the I-TASSER server [12-14]. I-TASSER reports the top five predictions for each submitted protein sequence, according to the c-score and the cluster size. We select the model that has the highest c-score out of the five returned models for each target sequence. I-TASSER also returns the top ten templates that it used for threading. We report the template that has the best sequence identity for the target protein sequence. For each unknown structure, Figure 3 shows the top prediction, the closest template, and the structural superposition of the predicted structure and the template. The c-scores for the models of YBR160W, YDL020C, and YML032C are 0.65, 0.41, and -0.54, respectively. We also compute the surface areas for each of the models and the reported template by using NACCESS which is an implementation of the methods described by Lee and Richards [29] and Hubbard, Campbell and Thornton [30]. The surface areas for the model for YBR160W and its template (PDB id 2PK9A) are 15,727Å^2^ and 15,074Å^2^, respectively which are similar. Also the surface areas of the model of YDL020C and its template (PDB id 1z1nx) are 33,655Å2 and 33,482Å2, respectively. The similarity in these surface areas can serve as a crude indication of the quality of the model returned from the server. In cluster 14, there are nine interactions. Four interactions involve YML032 whose model returned from the I-TASSER server is not a globular protein. This model is a very extended open structure. As a result, it would appear to have significant structural flexibility and thus not be fully suitable for rigid body docking using Cluspro. We have performed docking for the other five interactions. Results of docking for these five interactions are shown in Figure 4. For each interaction, the figure shows the surface views of the docked complexes. To measure how strongly these docked complexes are bound, we have calculated the buried surface area for each docked complex. Table 3 shows the buried surface area and the ratio between buried surface area and total surface area of each of the docked complexes.

**Table 3:** Buried surface area(SA) of the docked complexes

Interacting complex	Buried SA(Å^2^)	2*Buried SA/(Total SA)
Dimers		

YDL020C : YBR160W	5,603	0.23
YDL020C : YOL001W	4,517	0.20
YDL020C : YGL058W	4,295	0.20
YBR160W : YGL058W	3,408	0.29
YOL001W : YGL058W	2,162	0.21
YBR160W:YOL001W	5,779	0.41

Trimers		

YGL058W.YDL020C.YBR160W	9,898	0.34
YGL058W.YDL020C.YOL001W	8,812	0.33
YBR160W.YOL001W.YGL058W	7,941	0.44
YDL020C.YOL001W.YBR160W	10,296	0.33

Tetramers		

YGL058W.YDL020C.YBR160W.YDL020C	15,501	0.34
YGL058W.YDL020C.YOL001W.YBR160W	14,591	0.42

(the order of the complexes in this table is the same as in Figure 4(a–f) and Figure 5(a–d) for dimers and trimers, respectively)

YOL001W has 100% sequence identity with 2PK9 chain B and the template used by I-TASSER to predict the structure of YBR160W is 2PK9 chain A. This suggests that there might be an interaction between YOL001W and YBR160W because of this known dimeric structure. We docked the homolog (2PK9 chain A) of YOL001W and the model for YBR160W. The docked complex, YBR160W.YOL001W, is shown in Figure 4(f). The ratio of the buried surface area to the total surface area for this complex is the largest among all the dimers as shown in Table 3. Therefore if we consider buried surface area relative to the total surface area of a complex as a measure of the strength of an interaction between two proteins, the complex YBR160W.YOL001W is expected to be more stable than the other dimers. This could also mean that this new interaction between YBR160W and YOL001W would be stronger than the other pair-wise interactions.

It is evident from Figure 4(a), 4(b) and 4(c) that protein YDL020C has at least two binding sites. YBR160W and YOL001W both bind to YDL020C at overlapping sites but YGL058W binds with YDL020C at a completely different binding site. Thus, the interactions YDL020C.YBR160W and YDL020C.YGL058W or YDL020C.YOL001W and YDL020C.YGL058W could occur simultaneously. Figure 1(d) shows the new core of cluster 14 with YML032C and its related interactions removed and the newly discovered interaction YBR160W.YOL001W included. The docked complexes for these two set of mutually exclusive interactions, YGL058W.YDL020C.YBR160W and YGL058W.YDL020C.YOL001W, are shown in Figure 5(a) and 5(b) respectively. By analyzing the binding sites of YOL001W, we find that it has different binding sites to bind with YBR160W and YOL001W, thus making these two interactions concurrently possible. For a similar reason, the interactions YDL020C.YOL001W and YBR160W.YOL001W can occur simultaneously. The resultant trimers are shown in Figure 5(c)and 5(d), respectively. All other pair-wise interactions (4d, 4e and 4f) in Figure 4 are mutually exclusive because of shared binding sites of the interacting proteins. Table 3 shows the list of all possible higher order complexes that can be modeled from the four protein molecules in the new core (shown in Figure 1(d)) of cluster 14. We also compute the buried surface areas of the trimers, as shown in Table 3. This table also shows that the ratio between the buried surface area and total surface area for the trimer YBR160W.YOL001W.YGL058W is bigger than that of the other trimer thus making the former more stable. For similar reason, we rank the tetramer YGL058W.YDL020C.YOL001W.YBR160W as more stable than the tetramer YGL058W.YDL020C.YBR160W.YDL020C.  

**Figure 5:**
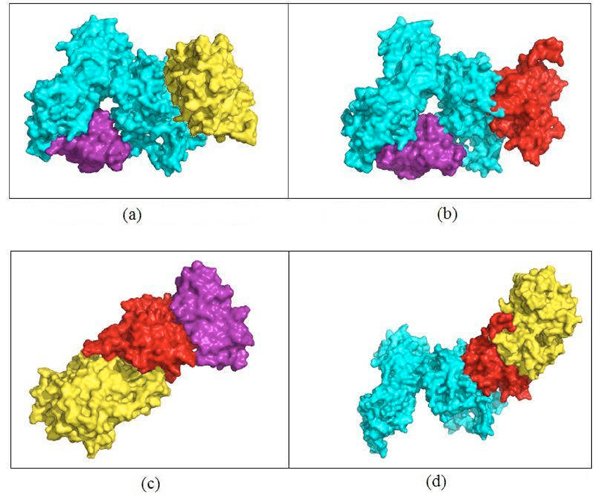
**Trimers built from pairs of interactions of proteins in the core of cluster 14.** Buried surface areas of the trimers (a) 9,898 Å^2^ for YGL058W.YDL020C.YBR160W (the docked complex if interactions a and c in Figure 4 occur simultaneously) (b) 8,812 Å^2^ for YGL058W.YDL020C.YOL001W (the docked complex if interactions b and c in Figure 4 occur simultaneously) (c) 7,941 Å^2^ for YBR160W.YOL001W.YGL058W (the docked complex if interactions e and f in Figure 4 occur simultaneously) (d) 10,296 Å^2^ for YDL020C.YOL001W. YBR160W (the docked complex if interactions b and f in Figure 4 occur simultaneously).

## Discussion

It is evident from the model for YML032C in Figure 3(c) that YML032C is a highly flexible protein. The results from disorder predictors [31] also show that this protein is disordered. High flexibility and disorder of this protein indicates that this could be a regulatory protein. Highly flexible and disordered proteins are functionally promiscuous as they can go through large and wide conformational changes while binding with other proteins or ligands [32]. Some disordered proteins attain tertiary structure of the binding site only when the binding with the ligand occurs. New methods that allow combining docking with folding of the disordered parts of a protein structure have been recently proposed [33-39]. Flexible docking can predict protein-protein interaction complexes while allowing limited flexibility of the interacting proteins. Most methods consider ligand flexibility [3,38,39] and some address hinge motion, side chain flexibility, and docking with multiple conformations of a target protein obtained from multiple structures for the same protein in the PDB database [38]. However no docking algorithm can presently treat the high flexibility and disorder as found in YML032C.

We have used the ratio between the buried surface area and the total surface area of a protein complex as a measure for the strength of an interaction. Although we cannot definitely say whether an interaction actually happens or not from the value of this ratio, the value itself gives a certain level of confidence in that interaction.

## Conclusion

This work has taken the approach of predicting new protein-protein interaction complexes and their interactions  through docking of their molecular structures. Since not all complexes are available in the PDB, nor are they all likely to ever be available, we have relied upon comparative modeling and docking methods. Their recent improved reliability gives some justification for the use of these approaches. This methodology has the advantage that it can also identify interactions that could occur together or ones that are mutually exclusive. In addition indirect interactions through another intermediate protein can be identified. However, because of the lengthy computational times and the required human judgment to select models from the results of the prediction programs for comparative modeling and docking, this process cannot yet be fully automated. Nonetheless many such cases can be investigated, and it appears that the results can provide important new information.

In this computational prediction of interaction complexes, new interactions, and concurrency or exclusiveness of multiple interactions require two major computational steps – comparative modeling (I-TASSER server [14]) and docking (Cluspro server [22,23]). We plan to develop software that will use a cluster of protein interactions as input to produce final structures.

Validation of these predictions is an important task. At this time, we have not experimentally validated these predictions of new protein-protein interactions and their complexes. Because of the relatively few structures for protein complexes in the PDB database, we have not found clusters where the structures for the predicted complexes are available in the PDB database. Therefore, at this point, the correctness of our results depends on that of the underlying computational methods – techniques for comparative modeling, clustering, and buried surface area computations. Our theoretical predictions might be however useful for crystallographers to select targets for the X-ray crystallographic determination of protein complexes.

## Competing interests

The authors declare that they have no competing interests.

## Authors’ contributions

ARK, AK, and RLJ all contributed to the design, execution and writing of this work.
